# A Practical Overview of the Articular Manifestations of Systemic Lupus Erythematosus

**DOI:** 10.7759/cureus.44964

**Published:** 2023-09-09

**Authors:** Juan Camilo Santacruz, Marta Juliana Mantilla, Sandra Pulido, Juan Ramón Isaza, Eduardo Tuta, Carlos Alberto Agudelo, John Londono

**Affiliations:** 1 Rheumatology Department, Comité de Estudios Médicos, Medellín, COL; 2 Rheumatology Department, Cireem IPS, Bogotá, COL; 3 Spondyloarthropathies Research Group, Universidad de La Sabana, Chía, COL

**Keywords:** treatment options, ultrasound, musculoskeletal manifestations, joint manifestations, systemic lupus erythematosus

## Abstract

Although it is widely known that joint involvement is the most frequent and prevalent manifestation of systemic lupus erythematosus (SLE), not having a validated organ-specific index for this domain in order to guide its treatment has been a major limitation. In addition, its clinical importance had been underestimated since it was not a vital risk domain; it was never the center of treatment, under the premise that in most cases its progression was slow and did not lead to significant functional disability. However, this concept has been changing due to the greater description of erosions both in ultrasonography and in osteoarticular magnetic resonance, so their identification can establish a more appropriate treatment time and thus avoid joint deformities, which in some cases can become irreversible. Recently, anifrolumab and belimumab have been able to significantly reduce the Systemic Lupus Erythematosus Disease Activity Index 2000 (SLEDAI-2K) and British Isles Lupus Assessment Group (BILAG) index scores, along with improvement in quality of life indices and a significant decrease in the required dose of glucocorticoids. Despite this, the ideal moment to consider biological therapy in this domain is not clear, since the clinical examination can sometimes be biased by the pain associated with fibromyalgia or the fatigue associated with SLE. For this reason, perhaps ultrasonography or magnetic resonance imaging, apart from differentiating the joint phenotype, can identify patients in time to define the onset of disease-modifying antirheumatic drugs and rationalize the use of glucocorticoids. The objective of this review is to characterize in detail the joint manifestations of SLE to offer the clinician a practical view of its diagnosis and treatment.

## Introduction and background

Systemic lupus erythematosus (SLE) is an autoimmune disease of variable severity, with a tendency to present flares in the course of its evolution. Immunological alterations, particularly the production of various antinuclear antibodies, are one of its determining characteristics, managing to affect any organ or system through the formation of immune complexes [1]. 

Amid the spectrum of articular manifestations tied to SLE, instances of inflammatory arthralgia take prominence, particularly affecting the joints of the hands and wrists, potentially progressing to overt synovitis. Jaccoud's arthropathy characterizes the malformative, reducible, and commonly non-erosive chronic arthritis linked with SLE, arising from capsular laxity, primarily affecting the metacarpophalangeal joints [2]. It should be noted that joint involvement is the most frequently observed clinical characteristic in SLE and can be present in close to 95% of cases [3]. Joint involvement, despite the fact that it is not a life-threatening condition, does lead to great functional disability and, therefore, adds a greater burden to the disease [4].

Joint symptoms are often the initial manifestations of lupus and may be present in up to 75% of patients at the time of diagnosis [5]. In accordance with established classification criteria, lupus-associated joint involvement is defined as the presence of synovitis in two or more joints, demonstrated by edema, pain, or joint effusion along with at least 30 minutes of morning stiffness [6]. Despite prior presumptions of non-erosive arthritis in SLE, advances in musculoskeletal ultrasound (US) and magnetic resonance imaging (MRI) have uncovered a higher prevalence of chronic synovitis and erosions than previously recognized [7]. It should be noted that joint involvement can manifest at any time after diagnosis. Musculoskeletal involvement is characterized by a wide variety of phenotypes and varying degrees of severity, ranging from minor arthralgias to erosive arthritis that can cause severe functional disability and reduced work productivity [8].

## Review

Clinical manifestations

Arthritis and Arthralgia (Non-Deforming)

Arthritis and arthralgia in SLE tend to be migratory and pain tends to disappear within 24 hours. Any type of joint can be affected and the inflammation tends to present in a symmetrical and polyarticular distribution involving the wrists, knees, and proximal interphalangeal joints. While the elbows, shoulders, and ankles can also be affected, their involvement is less frequent [9]. It's important to note that tendon-related issues (tenosynovitis) could be present in 10-44% of cases, underscoring the prevalence of conditions like rotator cuff syndrome, epicondylitis, Achilles tendonitis, and plantar fascia tendinitis [10,11].

Jaccoud's Arthropathy

**Figure 1:**
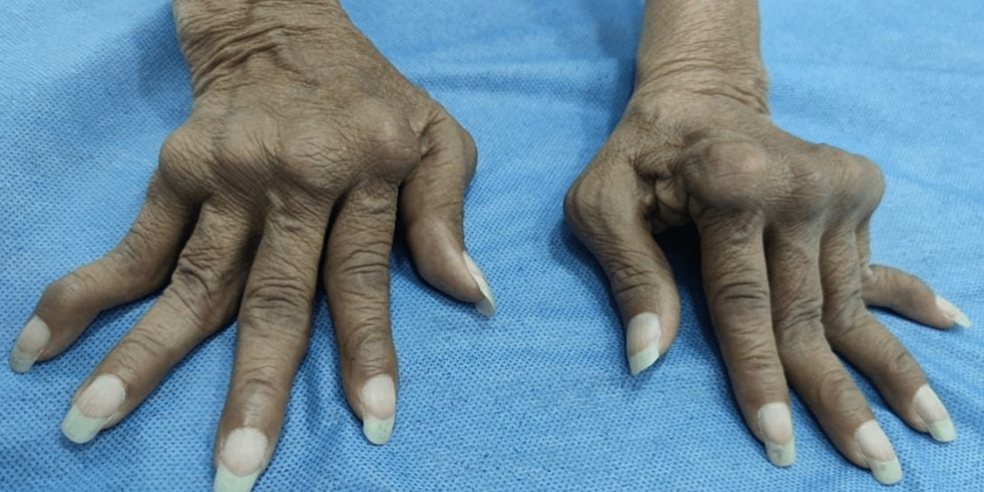
Jaccoud's arthropathy Deformities similar to those of rheumatoid arthritis can be seen such as ulnar deviation, swan neck deformity, and Z-thumb. Surgical central realignment of the extensor tendons of the metacarpophalangeal joints with joint stabilization will probably be required. Image Credit: Marta Juliana Mantilla, Rheumatologist

Despite previously being characterized as non-deforming, arthritis in SLE can exhibit deformities akin to those seen in rheumatoid arthritis (RA), encompassing ulnar deviation, buttonhole or swan neck deformities, Z thumb, hallux valgus, and various subluxations [12]. A prominent example is Jaccoud's arthropathy, a recurrent arthritis type capable of inducing deformities in the hands and feet, initially considered reversible, and resulting in erosions distinct from those seen in RA [13]. This disfiguring arthropathy was originally documented in patients with chronic rheumatic fever and recurrent arthritis episodes. In 1975, Bywaters coined the term "Jaccoud's arthropathy" to describe a similar condition in individuals with autoimmune disorders like SLE, Sjögren's syndrome, systemic sclerosis, and dermatomyositis [14-16]. This arthropathy has also been described in healthy older adults and in other diseases such as Parkinson's, some neoplasias, inflammatory bowel disease, and acquired immunodeficiency syndrome [17-19]. The deformities of Jaccoud's arthropathy are usually reducible and are attributed to the laxity of the joint capsules, tendons, and ligaments that cause joint instability, being the representation of a low-grade inflammatory process [20]. The exact prevalence of this type of arthropathy is unknown, but it has been reported that it may be close to 15%, being the second most frequent type of arthritis associated with SLE [21]. Risk factors associated with joint deformity include long-standing disease, the presence of anti-Ro and La antibodies, chronic use of glucocorticoids, spontaneous tendon rupture, and certain predisposing haplotypes such as human leukocyte antigen (HLA) A11 and B61 [22,23]. Figure 1 shows the classic findings of Jaccoud's arthropathy.

Rhupus

The term "Rhupus" has been used to describe patients with overlapping features of SLE and RA. The main feature of Rhupus is RA-like arthritis with lower lupus activity scores and less likely to present with major organ involvement such as lupus nephritis, neurologic manifestations, or hematologic abnormalities [24]. It has been reported that this syndrome is more common in women and, in most cases, it presents with the symptoms of RA initially and then progresses to the development of SLE within a period of four to seven years [25]. The most frequently reported clinical features are erosive polyarthritis, rheumatoid nodules, photosensitivity, alopecia, malar erythema, and constitutional symptoms. Some studies have shown that Rhupus patients have higher levels of human leukocyte antigen alleles DR1 and DR2 [26,27]. The prevalence of Rhupus among patients with SLE is highly variable in studies, ranging from 0.09% to 9.7%. The reasons for these discrepancies have been attributed to the inclusion criteria regarding the non-recognition of erosions as part of the entity, so it may still be underdiagnosed [28]. Patients with Rhupus and SLE maintain a similar prevalence of positivity for antinuclear antibodies (ANAs), anti-dsDNA, and anti-Smith (anti-Sm) [29]. 

Biomarkers

Patients with SLE generally have low rheumatoid factor (RF) titers in their serum without finding any relationship with the presence of erosive arthritis [30]. However, the positivity of anti-citrulline antibodies represents a 20-fold increase in the risk of developing erosions during the course of the disease [31]. In contrast, RF positivity in Jaccoud's arthropathy, along with antibodies directed against type II collagen, has been linked to deformity development [32]. Additionally, it has been postulated that elevated parathyroid hormone levels secondary to chronic renal failure or high-dose glucocorticoid administration might compromise the integrity of ligaments and tendons, leading to deformities through a direct impact on collagen formation [33]. A considerable number of patients with Jaccoud's arthropathy also exhibit antiphospholipid syndrome and valvular heart disease as concurrent conditions, not ruling out that small vessel vasculitis and immune complex deposition may contribute to periarticular fibrosis [34]. Table 1 shows the most representative differences between Jaccoud's arthropathy and Rhupus. 

**Table 1:** Most representative differences between Jaccoud arthropathy and Rhupus SLE: systemic lupus erythematosus

Jaccoud's arthropathy	Rhupus	References
Deformities are usually reducible	Initially presents as rheumatoid arthritis and then progresses to SLE in 4-7 years	[20,25]
The erosions occur late in time and are attributed to mechanical stress, induced by primary capsular-ligament involvement	The presence of erosions is a predominant feature	[20,24]
Rheumatoid factor positivity and the presence of antibodies against type 2 collagen are common	Anti-citrulline antibodies are present in high titers along with positivity for anti-RA-33 antibodies	[31,32]
High levels of human leukocyte antigen A11 and B61	Higher levels of human leukocyte antigen alleles DR1 and DR2	[22,26]

Diagnostic imaging

Conventional radiography was previously considered the gold standard for the evaluation of joint involvement in SLE, highlighting the presence of acral sclerosis, peri-articular osteopenia, soft tissue calcification, cystic lesions, and joint subluxation with bone erosions in cases of Jaccoud's arthropathy [35]. However, its diagnostic value has been lost because its sensitivity for demonstrating early structural changes in joints and soft tissues is low. Recent US studies have confirmed damage to other non-synovial structures such as the enthesis and tendons. Although it is known that up to 50% of patients with lupus report generalized myalgia, only 10% of patients present true inflammatory myositis [36]. A study evaluated the presence of US inflammation in 28 patients with lupus and arthralgia of the hands and wrists without clinical or previously documented arthritis, describing tenosynovitis in the extensor tendons of the fingers and active synovitis in 39.2% and 14.2%, respectively [37]. Another study supported the presence of inflammation in the US in 20 of 26 patients (76.9%) in patients with lupus and arthralgia without clinical synovitis. Synovial effusion was the most prevalent US findings, found in 50% of tendon structures and 34% of joint structures [38]. There are several studies that have evaluated the sensitivity of MRI in the hands and wrists to establish the presence of erosions. One of them evaluated 34 patients with or without evidence of synovitis or joint deformity, documenting the presence of erosions on the wrists and proximal metacarpophalangeal joints, in 93% and 61% of patients respectively [39]. It has also been described that enthesitis is more prevalent in patients with SLE where the most compromised enthesis sites are the tibial insertion of the patellar tendon followed by the calcaneal insertion of the Achilles tendon [40].

Treatment

Currently, the choice of treatment is based on validated clinical activity indices such as the Systemic Lupus Erythematosus Disease Activity Index 2000 (SLEDAI-2K) or the British Isles Lupus Assessment Group (BILAG), musculoskeletal being highly weighted by the presence of synovitis [41,42]. All lupus patients should start treatment with hydroxychloroquine at diagnosis unless contraindicated as it has been shown to be effective in controlling joint symptoms and preventing disease flare-ups [43]. If clinical response is not achieved three months after initiation, a course of non-steroidal anti-inflammatory drugs (NSAIDs) at the lowest dose and shortest duration possible may be considered, particularly in patients with lupus nephritis or high cardiovascular risk [44]. Patients who are contraindicated to NSAIDs or do not respond to them are considered a short course of glucocorticoids (either orally or intramuscularly) for two to four weeks depending on clinical response. In cases where glucocorticoid continuation is required for more than one month, a disease-modifying antirheumatic drug (DMARD) should be added, preferably methotrexate, to achieve glucocorticoid reduction and thus avoid its adverse effects [45,46]. Patients persistently experiencing joint activity despite methotrexate treatment over three to six months should undergo assessment for potential alternative therapeutic strategies. Such options encompass belimumab, anifrolumab, rituximab, azathioprine, or abatacept [47,48].

Belimumab

Belimumab is a human monoclonal antibody that inhibits the soluble form of B cell survival factor (BLyS), demonstrating its usefulness for patients with SLE whose articular and cutaneous manifestations present predominantly. Their clinical trials have shown a great improvement in the control of musculoskeletal symptoms, also achieving a lower requirement of glucocorticoids [49,50]. In a Cochrane review that encompassed six clinical trials, it was reported that belimumab, either alone or in combination with other immunosuppressive drugs, reduces SLEDAI 2K disease activity with a relative improvement compared to placebo of 13%, with statistical significance [51].

Anifrolumab

The therapeutic benefit of inhibiting the interferon pathway in patients with SLE has been established in several clinical trials. Anifrolumab, a fully human IgG1κ monoclonal antibody against type I interferon receptor subunit 1, has been shown to stabilize persistent joint symptoms, improve composite indices of SLE activity, and allow glucocorticoid dose reduction [52]. The included patients were classified as having moderate to severe activity despite standard treatment, excluding patients with lupus nephritis and neuropsychiatric disease [53,54]. In a post hoc analysis, a higher proportion of patients receiving anifrolumab (56.7%) demonstrated near-complete resolution of arthritis by SLEDAI 2K, also achieving favorable results in terms of joint domain by BILAG [55].

Azathioprine

The efficacy of azathioprine has been extrapolated from its evidence in RA as well as the effect it has to improve certain hematological, gastrointestinal, and neurological manifestations of lupus. Azathioprine has been shown to lower the SLEDAI-2K score with the consideration that it may stabilize some musculoskeletal manifestations of SLE. However, it is preferable that its initiation not be considered solely for the control of joint manifestations given the scant evidence that exists to date, and its initiation would be justified if it is used to control another extra-renal manifestation of SLE [56-58].

Rituximab, Abatacept, and Baricitinib

Evidence for rituximab is largely based on observational studies showing a reduction of lupus activity by SLEDAI or BILAG [59]. A singular clinical trial focused on SLE patients treated with abatacept examined occurrences of arthritis, discoid lesions, and pleurisy. The trial revealed a decrease in the frequency of BILAG-defined arthritis flares, though not for discoid lesions [60]. In a separate phase 2 trial involving 314 patients with SLE presenting skin and joint manifestations, the efficacy of baricitinib was examined at doses of 4 mg/day and 2 mg/day at the 24-week mark. The 4 mg dose exhibited improvements in joint symptoms and SLEDAI 2K-associated rash among patients who had not achieved control of these manifestations using conventional therapy, whereas the 2mg dose did not yield similar outcomes [61].

**Figure 2:**
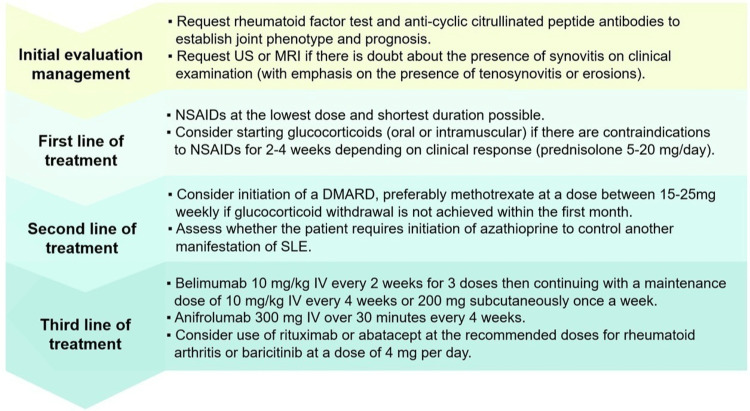
Therapeutic approach for patients with SLE with joint involvement DMARD: disease-modifying antirheumatic drug; IV: intravenous; MRI: magnetic resonance imaging; NSAIDs: Non-steroidal anti-inflammatory drugs; SLE: systemic lupus erythematosus; US: ultrasound References: [44-48]

Figure 2 describes the therapeutic approach for patients with SLE with joint involvement according to the available levels of evidence.

Joint Replacement Surgery

Total arthroplasty of certain joint groups is sometimes required in some patients with SLE. In a British cohort of 500 patients, only 19 patients (4%) required a complete joint replacement over a 30-year follow-up period. In advanced cases of Jaccoud's arthropathy, when the deformities are fixed and irreversible, surgical surgery on the subluxated bones or soft tissues may be required [62,63]. It should be noted that surgical treatments can fail in up to 70% of cases, although arthroplasties performed by expert surgeons can achieve favorable results in slightly more than half of the patients who undergo the procedure [64].

Prognosis

The prognosis of joint involvement will result from the individual clinical phenotype. Arthralgias and non-disfiguring arthritis do not generally carry significant functional disability, although clinically establishing inflammatory joint involvement can sometimes be challenging given that musculoskeletal pain, fatigue, and stiffness are also characteristics of fibromyalgia [65]. Although Jaccoud's arthropathy in its initial stages behaves like a non-erosive deforming arthritis, it is possible that at some point the inflammatory process may cause erosions, requiring in some patients the initiation of conventional or biological DMARD to prevent the progression of deformities, although the timing of its onset is unknown [66]. The presence of anti-citrulline and anti-RA-33 antibodies may be useful to differentiate erosive arthritis of SLE or Rhupus from Jaccoud's arthropathy, thus having prognostic implications [67]. In general, musculoskeletal damage is severe in Rhupus, being similar to that of RA, although the correlation between the number of erosions and their impact on functional disability is unknown [68].

## Conclusions

Inflammatory joint involvement in SLE, although being the most common manifestation of the disease, lacks a definitive and specific measure to guide therapeutic responses. The use of SLEDAI and BILAG could overestimate joint activity leading to the need to restart glucocorticoids and maintain them for a long time, favoring the accumulated damage associated with their chronic use. Additionally, there are many confounding variables at the time of clinical evaluation to determine whether or not the presence of synovitis is clear, such as nociplastic pain associated with fibromyalgia or arthralgia associated with fatigue.

The routine use of US and MRI is increasingly favored to establish the clinical phenotype of joint involvement and more clearly define its prognosis. Confirming inflammatory findings in both US and MRI could more objectively and rationally justify the use of biological therapy for this domain, highlighting belimumab and anifrolumab for their current evidence and better safety profile. It is necessary not to overlook the fact that cases of Jaccoud's arthropathy can progress over time to present irreversible deformities, just like Rhupus, so timely treatment could prevent functional damage, preserving the quality of life.
